# Adjuvant choice shapes macrophage activation and cytokine responses to freeze–thaw breast cancer antigens

**DOI:** 10.1186/s13104-026-07840-4

**Published:** 2026-04-26

**Authors:** Murat Ihlamur, Emrah Şefik Abamor

**Affiliations:** 1 Department of Bioengineering, Faculty of Chemical and Metallurgical Engineering, Yildiz Technical University, Istanbul, Turkey; 2 Department of Electronics and Automation, Vocational School, Biruni University, Istanbul, Turkey

**Keywords:** Breast cancer, Immunotherapy, Tumor antigens, Adjuvants, Cancer vaccines

## Abstract

**Objective:**

To investigate how different adjuvants influence macrophage activation induced by freeze–thaw whole-cell lysates prepared from two breast cancer cell lines (MCF-7 and MDA-MB-231), we evaluated the effects of alum and saponin in J774 macrophages by assessing cell viability, nitric oxide (NO) production, and cytokine responses.

**Results:**

Freeze–thaw breast cancer antigens (FTBA) triggered macrophage responses in a dose-dependent manner, with 40 µg/mL providing a suitable balance between activation and viability. When combined with adjuvants, FTBA + alum maintained higher macrophage viability compared with FTBA + saponin. Alum-adjuvanted FTBA induced stronger NO production than saponin-adjuvanted formulations. Cytokine profiling showed that alum-adjuvanted FTBA induced the strongest cytokine responses overall, with fold-increases in IL-6, IL-12, TNF-α, and GM-CSF observed for both antigen sources. Overall, alum enhanced macrophage activation with lower cytotoxicity than saponin, indicating that adjuvant selection significantly modulates innate immune responses to freeze–thaw tumor antigens.

**Graphical Abstract:**

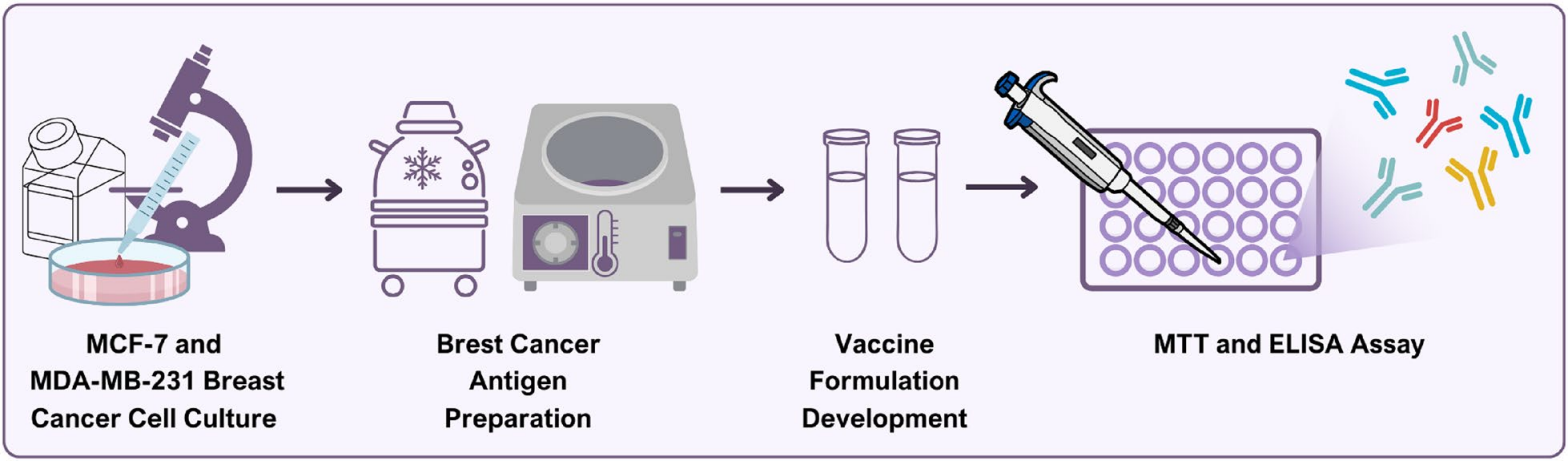

## Introduction

Breast cancer remains one of the leading causes of cancer-related morbidity and mortality worldwide [1, 2]. Whole-cell tumor lysates provide a broad antigen repertoire and can be produced rapidly, making them attractive as vaccine antigen sources [3–5]. However, the innate immune activation triggered by tumor lysates is highly dependent on adjuvant selection [6–8].

Macrophages are highly relevant to breast cancer biology because they are among the first immune cells to respond to tumor-derived signals and can influence inflammatory signaling, antigen processing, and subsequent immune activation. Freeze–thaw whole-cell lysates are not only a practical source of broad tumor-associated antigens, but may also contain intracellular danger signals released during cell disruption. Therefore, evaluating macrophage responses to freeze–thaw breast cancer antigens provides a useful initial model for understanding how lysate-based vaccine formulations may shape early innate immune responses in a breast cancer-related context.

In this study, freeze–thaw breast cancer antigens (FTBA) were generated from two breast cancer subtypes (MCF-7 and MDA-MB-231) and tested on J774 macrophages. The study was designed as a comparative in vitro screening approach to evaluate adjuvant-dependent macrophage responses under controlled conditions, rather than as a direct representation of the human breast tumor microenvironment. We first screened FTBA dose ranges to identify a working antigen dose, then evaluated alum and saponin dose–response combinations with the selected antigen concentration. Macrophage responses were assessed by cell viability (MTT), nitric oxide (NO) release, and cytokine secretion (IL-6, IL-12, TNF-α, GM-CSF) to compare how adjuvant choice shapes the inflammatory profile induced by FTBA.

## Materials and methods

### Materials

FBS, RPMI-1640, DMEM, penicillin–streptomycin, L-glutamine, MTT reagent, and DMSO were used for cell culture and viability analysis. Alum and saponin were used as adjuvants. Cytokine ELISA kits for IL-6, IL-12, TNF-α, and GM-CSF were obtained from BTLAB (China).

### Breast cancer cell cultures

MCF-7 and MDA-MB-231 human breast cancer cell lines were cultured under standard conditions (37 °C, 5% CO₂) in appropriate complete media supplemented with 10% FBS and 1% penicillin–streptomycin [9].

### Preparation of breast cancer antigens by the freeze–thaw method

Breast cancer antigens were prepared from MCF-7 and MDA-MB-231 cell lines using the freeze–thaw technique, beginning with the thawing of previously frozen PBS-suspended cells (stored at − 20 °C) in a 37 °C water bath [10]. The cells were subsequently subjected to freezing in liquid nitrogen for 15 min. Subsequently, the samples underwent a second thawing phase in a 37 °C water bath for 15 min. To achieve thorough cellular disruption, the freeze–thaw cycle—alternating between liquid nitrogen freezing and water bath thawing—was repeated five times. Following the final cycle, the resulting lysates were centrifuged at 10,000 rpm for 3 min to separate cellular debris. The supernatant, containing the soluble antigenic components, was carefully collected [11]. Protein concentration was measured using a standard protein quantification method, and lysates were stored at − 20 °C until use.

### Development of vaccine formulations

FTBA was first tested alone at 10, 20, 40, 80, 100, and 160 µg/mL to identify a working antigen dose based on macrophage viability and NO output. The tested concentrations were selected to cover a broad range of antigen exposure, and 160 µg/mL was included as the highest concentration to assess responses under upper-range conditions. After selecting 40 µg/mL as the working FTBA concentration, adjuvant dose–response experiments were performed by combining FTBA (40 µg/mL) with alum or saponin at 10–160 µg/mL. Based on viability and NO results, 40 µg/mL FTBA + 40 µg/mL adjuvant was chosen for downstream cytokine testing.

### Nitric oxide (NO) analysis

NO production was measured in culture supernatants using the Griess assay. J774 macrophages were stimulated with FTBA alone (10–160 µg/mL) or with FTBA fixed at 40 µg/mL combined with alum or saponin (10–160 µg/mL). After incubation, absorbance was measured at 540 nm and NO levels were calculated from a nitrite standard curve [12].

### Cell viability assay

J774 macrophages were seeded at 1 × 10⁴ cells/well in 96-well plates and incubated for 24 h. Cells were then treated with FTBA formulations and incubated for an additional 48 h. Viability was determined using the MTT assay and absorbance was read at 570 nm. Viability (%) was calculated relative to untreated control [13].

### In vitro quantification of cytokine levels

Cytokines (IL-6, IL-12, TNF-α, GM-CSF) were quantified in macrophage supernatants using commercial ELISA kits (BTLAB, China) according to the manufacturer’s instructions. For cytokine experiments, the following groups were tested: control, FTBA (40 µg/mL) alone, FTBA (40 µg/mL) + alum (40 µg/mL), and FTBA (40 µg/mL) + saponin (40 µg/mL).

### Statistical analysis

Data are presented as mean ± SD. Statistical comparisons were performed using one-way ANOVA with Tukey’s post-hoc test. Experiments were repeated in three independent biological replicates, each measured in technical triplicate. A p value < 0.05 was considered statistically significant.

## Results

### Cell viability analyses

**Fig. 1:**
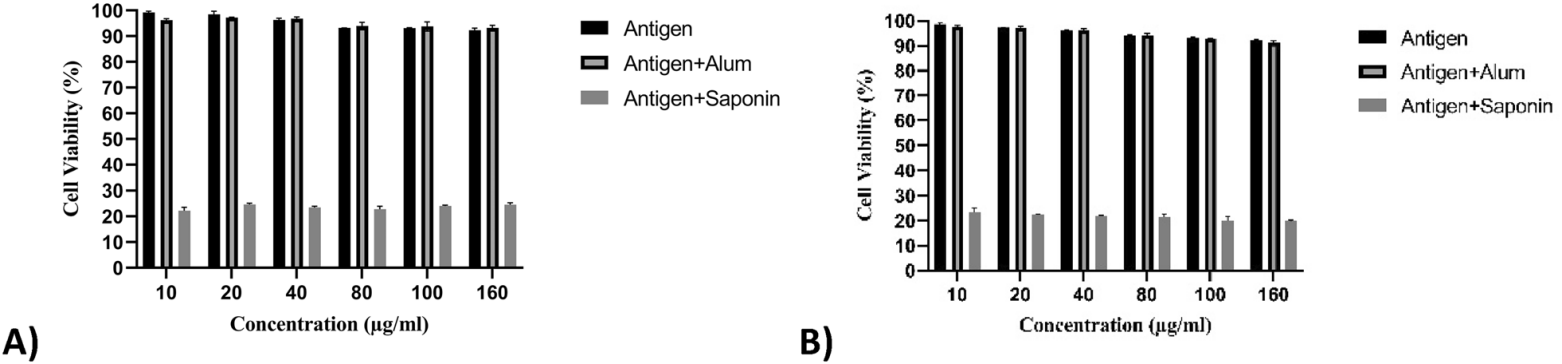
Viability of J774 macrophages after exposure to freeze–thaw breast cancer antigens (FTBA) derived from **A** MCF-7 and **B** MDA-MB-231 cells at different antigen concentrations. This initial concentration screening was used to determine the antigen dose for subsequent adjuvant combination experiments. Cell viability was assessed by MTT assay. Data are presented as mean ± SD. Statistical significance is indicated in the figure

FTBA induced dose-dependent effects on J774 viability, and 40 µg/mL was selected as the working antigen concentration. When FTBA (40 µg/mL) was combined with adjuvants, alum maintained high viability (~ 96%), whereas saponin caused marked cytotoxicity (~ 20–25% viability) in both MCF-7- and MDA-MB-231-derived formulations (Fig. 1).

### Nitric oxide (NO) production

**Fig. 2:**
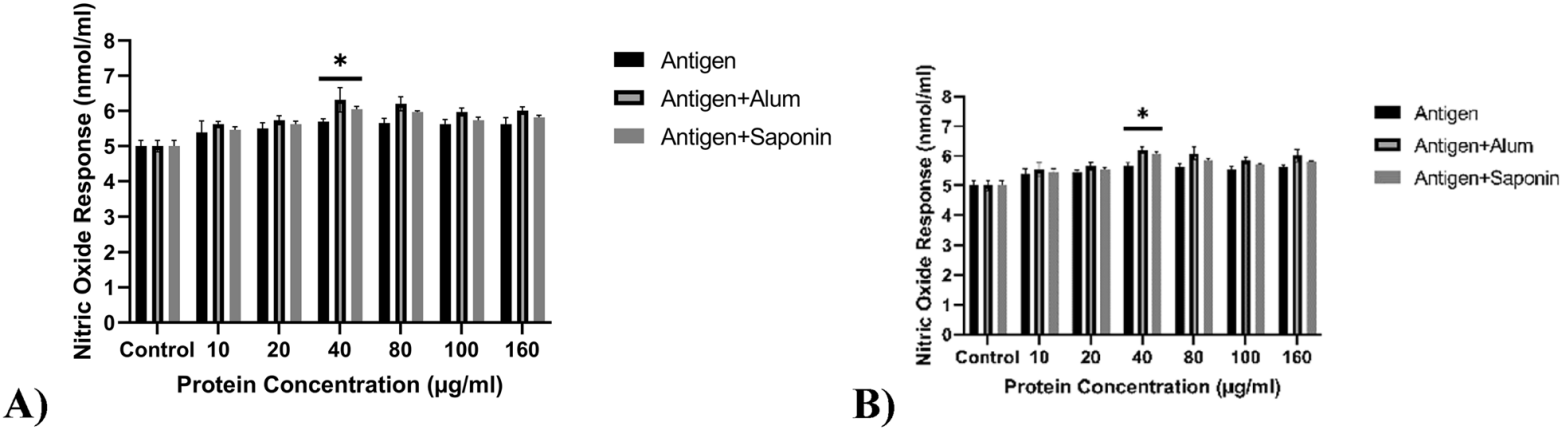
Nitric oxide (NO) production by J774 macrophages stimulated with freeze–thaw breast cancer antigens (FTBA) derived from **A** MCF-7 or **B** MDA-MB-231 cells in combination with alum or saponin at different adjuvant concentrations. Based on the preliminary antigen screening, FTBA was fixed at 40 µg/mL for these experiments, and NO production was measured by the Griess assay. Data are presented as mean ± SD. Statistical significance is indicated in the figure

FTBA stimulation increased NO production in J774 macrophages, with peak responses observed around the 40 µg/mL antigen dose. Using FTBA at 40 µg/mL, co-administration with alum further increased NO levels compared with antigen alone, whereas saponin produced only a limited NO increase in the context of substantial cytotoxicity (Fig. 2).

### Cytokine production

**Fig. 3:**
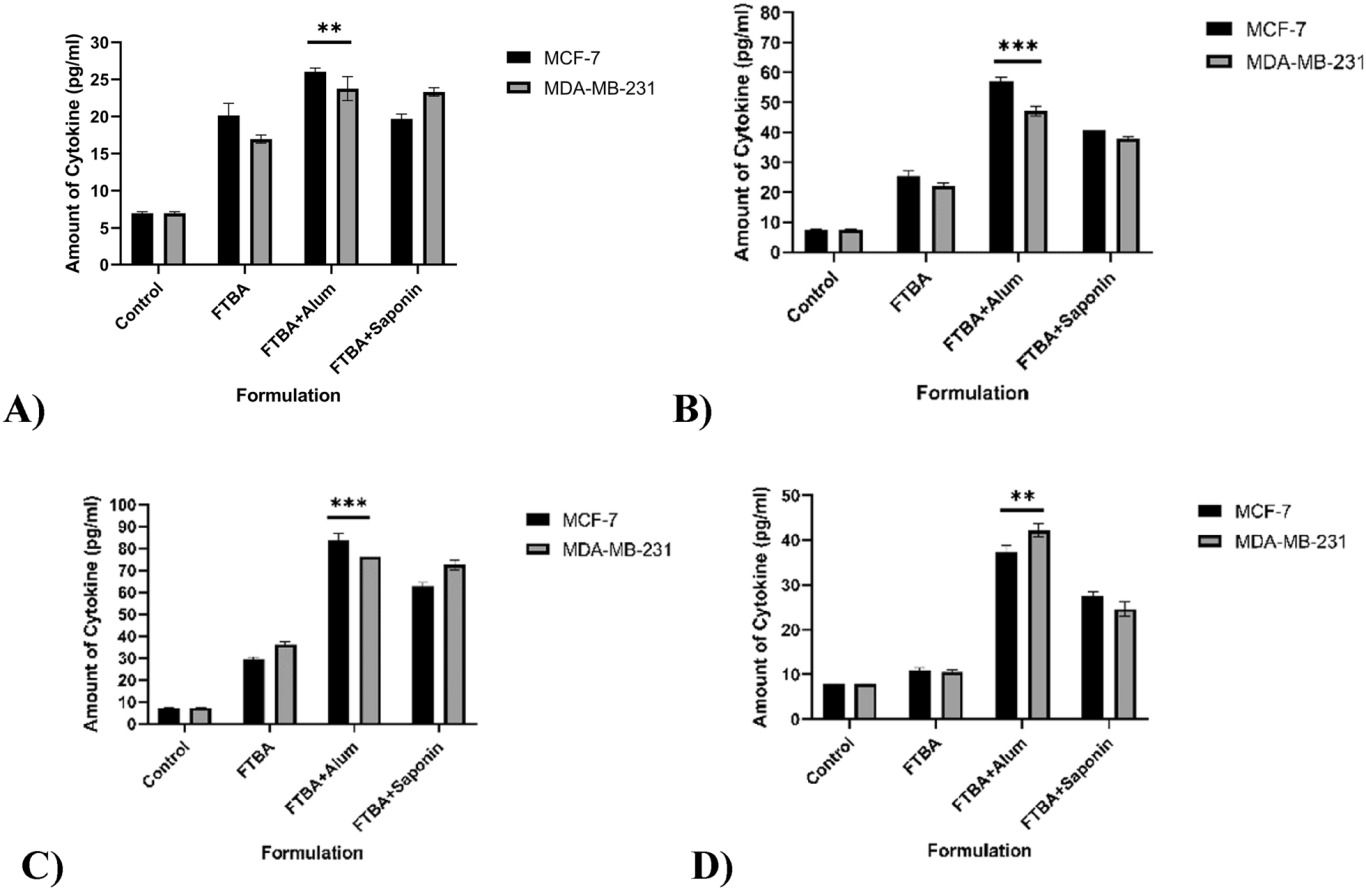
Cytokine responses of J774 macrophages following stimulation with freeze–thaw breast cancer antigen (FTBA) formulations. Supernatant levels of **A** IL-6, **B** IL-12, **C** TNF-α, and **D** GM-CSF were measured in control, FTBA alone, FTBA + alum, and FTBA + saponin groups. Based on the preliminary screening experiments, FTBA was used at 40 µg/mL, and adjuvants were also evaluated at 40 µg/mL. Data are presented as mean ± SD. Statistical significance is indicated in the figure

Cytokine secretion (IL-6, IL-12, TNF-α, and GM-CSF) was evaluated in control, FTBA alone (40 µg/mL), FTBA + alum (40 µg/mL), and FTBA + saponin (40 µg/mL) groups (Fig. 3). FTBA alone increased cytokine production relative to the untreated control, indicating that freeze–thaw breast cancer lysates were sufficient to induce measurable macrophage activation. Among the tested formulations, FTBA + alum consistently produced the strongest cytokine responses for both antigen sources.

For MCF-7-derived FTBA + alum, IL-6, IL-12, TNF-α, and GM-CSF increased by 3.72-, 7.609-, 11.3-, and 4.77-fold, respectively, compared with the control group (*p* < 0.01–0.001). For MDA-MB-231-derived FTBA + alum, the corresponding increases were 3.41-, 6.302-, 10.3-, and 5.41-fold, respectively, compared with the control group (*p* < 0.01–0.001). These findings suggest that alum supported a stronger and more measurable pro-inflammatory cytokine response under the tested conditions. Although FTBA + saponin also showed inflammatory activity, interpretation of this group is limited by the reduced cell viability observed at the same concentration.

## Discussion

This study shows that freeze–thaw breast cancer lysates activate macrophages in a dose-dependent manner, and 40 µg/mL was a practical working concentration for downstream formulation studies. When combined with adjuvants, alum preserved cell viability while enhancing NO release and strongly increasing pro-inflammatory cytokines, supporting its suitability for lysate-based vaccine formulations [14–16]. In contrast, saponin at the tested concentrations caused marked cytotoxicity in J774 macrophages, which limits interpretation of its immunostimulatory profile in this in-vitro setting [17–19]. Saponin-based adjuvants can promote cross-presentation and CD8⁺ T-cell responses, although tolerability may depend on formulation and dose [20, 21]. These findings highlight that adjuvant selection critically determines both the magnitude and interpretability of macrophage responses to tumor lysate antigens. The differences between MCF-7- and MDA-MB-231-derived lysates further suggest that tumor subtype may influence innate immune activation. The cytokine profile observed with alum-adjuvanted FTBA suggests that this formulation may provide a more interpretable innate activation pattern during early antigen encounter, at least under the in vitro conditions tested here.

The 40 µg/mL concentration used for alum and saponin was intended for standardized in vitro comparison and is not directly comparable to adjuvant doses used in patients, which depend on formulation, route, and in vivo distribution. Therefore, these findings should be interpreted as comparative screening data rather than a direct indicator of clinical dose performance. Although alum- and saponin-associated immune activation has been linked in previous studies to signaling pathways such as NF-κB, MAPK, and inflammasome-related mechanisms, the present findings are limited to functional outputs measured by viability and cytokine release. Therefore, the observed differences between adjuvant groups should not be interpreted as direct evidence of pathway-specific activation. Further studies using transcriptional or protein-level analyses will be needed to define the signaling mechanisms underlying these responses.

### Limitations

This study is restricted to a single murine macrophage cell line (J774) and in vitro readouts. Therefore, the findings should be interpreted as comparative in vitro screening data rather than direct evidence of macrophage behavior in human breast cancer. The model may not fully represent primary human macrophage responses. In addition, the study did not include phenotypic or molecular analyses such as qPCR, Western blotting, or inflammasome-focused assays; therefore, possible involvement of NF-κB, MAPK, or NLRP3-related signaling could not be determined. In addition, the use of freeze–thaw lysates prepared from established breast cancer cell lines provides a practical and reproducible antigen source, but does not reflect the heterogeneity of patient-derived tumors. Likewise, the simplified monoculture system does not reproduce the biochemical and cellular complexity of the breast tumor microenvironment; therefore, the implications for breast cancer progression remain limited. Potential endotoxin contamination of whole-cell lysates cannot be fully excluded and may influence macrophage activation; future studies should incorporate endotoxin testing and appropriate neutralization controls. Finally, while alum favored robust cytokine release with minimal toxicity under the tested conditions, alum’s Th2-biased profile may limit T-cell-mediated anti-tumor responses, and cytokine interpretation for the saponin group is limited by the low cell viability observed under these conditions [22].

## Conclusion

Freeze–thaw breast cancer lysates activated macrophages in a dose-dependent manner, and 40 µg/mL provided a practical working antigen concentration. Among the tested adjuvants, alum preserved macrophage viability while enhancing NO release and pro-inflammatory cytokine secretion, whereas saponin caused marked cytotoxicity under the conditions tested. These findings highlight that adjuvant choice critically shapes the magnitude and interpretability of innate immune responses to tumor lysate antigens and support alum-adjuvanted FTBA as a rational formulation for further in vivo evaluation [23].

**Learning Points**.

- FTBAs at 40 µg/mL activated macrophages without inducing cytotoxicity.
- FTBA plus alum increased NO and the cytokines IL-6, IL-12, TNF-α, and GM-CSF while preserving cell viability.
- Despite boosting cytokine release, saponin-containing formulations caused marked cytotoxicity; adjuvant choice strongly shapes the immune response.
- Response magnitude differed between lysates from luminal A (MCF-7) and triple-negative (MDA-MB-231) cells, suggesting tumor-subtype-dependent antigenic content.
- Overall, breast tumor lysate + alum appears to be a rational vaccine platform warranting in vivo evaluation.

## Data Availability

The datasets used and/or analysed during the current study are available from the corresponding author on reasonable request.

## Abbreviations

DMEM: Dulbecco’s Modified Eagle Medium
RPMI-1640: Roswell Park Memorial Institute medium 1640
DMSO: Dimethyl sulfoxide
ELISA: Enzyme-linked immunosorbent assay
FBS: Fetal bovine serum
FTBA: Freeze–thaw-based (whole-cell) antigen
GM-CSF: Granulocyte–macrophage colony-stimulating factor
IL-6: Interleukin-6
IL-12: Interleukin-12
J774: Murine macrophage cell line
MCF-7: Human breast cancer cell line (luminal A)
MDA-MB-231: Human breast cancer cell line (triple-negative)
MTT: 3-(4,5-Dimethylthiazol-2-yl)-2,5-diphenyltetrazolium bromide
NO: Nitric oxide
PBS: Phosphate-buffered saline
TNF-α: Tumor necrosis factor-alpha
